# Design and implementation of the participatory German network for translational dementia care research (TaNDem): A mixed‐method study on the perspectives of healthcare providers and dementia researchers in dementia care research

**DOI:** 10.1111/hex.13748

**Published:** 2023-03-12

**Authors:** Annelie Scharf, Anika Rädke, Daniel Purwins, Marie Marleen Heppner, Stefanie Köhler, Martina Roes, Stefan Teipel, Wolfgang Hoffmann, Bernhard Michalowsky

**Affiliations:** ^1^ Deutsches Zentrum für Neurodegenerative Erkrankungen (DZNE), Rostock/Greifswald Greifswald Germany; ^2^ Deutsches Zentrum für Neurodegenerative Erkrankungen (DZNE) Witten Germany; ^3^ Department of Nursing Science, Faculty of Health University of Witten/Herdecke Witten Germany; ^4^ Section for Gerontopsychosomatics and Dementia‐Related Diseases, University Medical Centre Rostock Clinic and Polyclinic for Psychosomatic Medicine and Psychotherapy Rostock Germany; ^5^ Section Epidemiology of Health Care and Community Health, Institute for Community Medicine University Medicine Greifswald (UMG) Greifswald Germany

**Keywords:** Alzheimer's disease, dementia, dementia care research, network, participatory research, stakeholder involvement

## Abstract

**Background:**

Currently, there is a lack of interaction between research and healthcare practice. As a result, research findings reach healthcare practice only late, and topics relevant to practice are often not known in research. Involving people living with dementia (PlwD), their relatives and healthcare providers in dementia care research can accelerate this process. For inclusion, firm and reliable structures are needed, which are to be established with the help of the Translational Network for Dementia Care Research in Germany. However, there is only limited knowledge about the priorities, expectations and conditions of stakeholders (healthcare providers and dementia researchers) for such cooperation within a network.

**Objectives:**

The aim is to gather stakeholders' views on (i) future research topics to be addressed within the dementia care research network, (ii) the nature of collaboration within the network and (iii) the facilitating and hindering factors for establishing such a network.

**Methods:**

Within an exploratory sequential mixed‐method study, we interviewed 87 stakeholders within eleven semistructured focus group interviews. The interviews were transcribed, pseudonymized and analyzed using qualitative content analysis. The qualitative data were analyzed with MAXQDA. Based on the qualitative results found in the focus group interviews, a supplementary online questionnaire was developed to prioritise and rank these findings afterwards.

**Results:**

Stakeholders prioritized a comprehensible transfer of research results into practice, increased involvement of PlwD and their relatives (additionally marginalized groups such as people with a migrant background) in research and exchange between researchers. Cooperation should preferably occur in a regional context with local contacts, and the latest research results should be made available via an online database. The stakeholders' time, finances and human resources should be considered.

**Conclusion:**

Stakeholders have partly similar preferences and goals for cooperation and involvement, emphasizing that such interaction in a network offers the possibility of long‐term, effective collaboration and added value for practice and research.

**Patient or Public Contribution:**

For this study, dementia healthcare providers and dementia care researchers were asked about their perspectives. Their involvement is further elucidated in the manuscript text.

## 1 INTRODUCTION

Population ageing is one of the challenges health systems face globally. This is associated with an increase in the prevalence of people living with dementia (PlwD).^1^ The World Alzheimer Report 2021 estimated that more than 55 million people live with dementia worldwide. According to forecasts, the number of people affected will rise to 78 million by 2030.^2^

These developments have led to an increased demand for healthcare services, which pose new challenges for managing treatment and care.^3^ Consequently, the need for studies evaluating the efficacy and effectiveness of treatment options has increased steadily over the last 50 years. However, it became apparent that due to the dominance of researchers and the lack of interaction between researchers and healthcare providers, there was a discrepancy between the dementia research topics that researchers prioritized and those that were needed in practice.^4^ Another problem is the failure to translate research into practice and policy. As a result, it is impossible to ensure that effective and cost‐efficient programmes, services and medicines reach all who need them, and health professionals cannot achieve the level of care they aspire to. Consequently, the interest in transferring knowledge into practice has increased drastically.^5^

Participatory research aims to actively involve people directly or indirectly affected by a disease such as dementia in research and to work with them as partners instead of considering them only as participants.^6^ A participatory approach offers the possibility of linking scientific and practical perspectives.^7^ Public participation in research is essential to support the development of care management solutions.^3^ The involvement of the general public (‘patients, potential patients, carers and people who use health and social care services as well as people from organisations that represent people who use services’^6^) is desirable both for methodological reasons, as this leads to higher‐quality research with greater impact, and for ethical reasons, as people affected by research have a right to have a say in what and how research is conducted.^8^

In the United Kingdom^9^ and Canada,^10^ participatory approaches have already been implemented in dementia research. A list of key dementia research topics has been developed through interviews with PlwD, their carers/relatives and health and social care providers to set up the dementia research agenda jointly. The Scottish Dementia Clinical Research Network also surveyed PlwD and their carers, researchers and the general public interested in dementia research about their priorities for dementia research, preferred types of research, desired outcome parameters and willingness to volunteer. The top four topics were: Early detection of dementia, drug studies, studies on people living at home and caregivers.^11^

A German study^3^ identified and analyzed research priorities and views on participation in research amongst 31 healthy seniors and 16 family and formal caregivers. Results showed that the main interests of both groups, including early detection of dementia, person‐centred care and the involvement of relatives in research, were largely similar. In addition to this research, other groups of people relevant to dementia research and interested in dementia care need to be identified and interviewed as part of future research about their willingness to participate in dementia care research.^3^ Likewise, another study looked at the challenges of working across networks due to differences between Canadian provinces and highlighted the need for consistent and collaborative working between individual health research and healthcare providers.^12^

So far, evidence is limited on the most effective methods of involving the public in health and social care research.^13^ This evidence needs to be strengthened significantly.^14^, ^15^ Despite the list of research priorities in dementia care published by the World Health Organization, which explicitly emphasizes the perspectives of researchers and stakeholders,^16^ there is a lack of studies on research priorities in dementia research in the context of country‐specific network structures.^3^ To our knowledge, only one study exists in Germany,^3^ of which surveyed 16 healthcare providers about their research priorities. Furthermore, the perspective of healthcare providers as a separate group is rarely addressed in existing studies.^3^ Therefore, the evidence around healthcare providers' views needs to be significantly expanded. Furthermore, strategies are required to enable sustainable cooperation in a national context because various research results^17^, ^18^, ^19^, ^20^, ^21^, ^22^ show that measures for better coordination of dementia care can be a meaningful instrument in healthcare and can sustainably improve the health and well‐being of those affected.

For these reasons, the German Centre for Neurodegenerative Diseases (DZNE) has set itself the goal to establish a Translational Network for Dementia Care Research (TaNDem) in Germany by incorporating the perspectives of PlwD, their relatives, healthcare providers and dementia researchers. In preparation for this network, we investigated the views of stakeholders (healthcare providers and dementia researchers) on (i) important and unaddressed research topics, (ii) the nature of network collaboration and (iii) supporting and hindering factors of network implementation and sustainability. This article focuses on the perspectives of dementia researchers and healthcare providers. The views of PlwD and their carers will be the focus of a separate paper. Due to the abundance of findings from the interviews, it is clearer to present the results separately.

## 2 METHODS

### 2.1 Study design and sample

**Figure 1:**
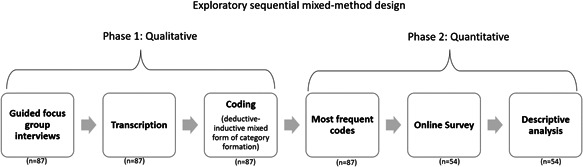
A flowchart of the study procedures: Exploratory sequential mixed‐method design.

We conducted an exploratory sequential mixed‐method study (Figure 1) consisting of qualitative focus group interviews followed by the development of a quantitative online survey to prioritize the findings. preferences for participation in research and participatory collaboration in a dementia care research network. Within the TaNDem network, the participating DZNE sites Berlin, Bonn, Dresden, Goettingen, Magdeburg, Rostock/Greifswald, Ulm and Witten contacted dementia researchers and healthcare providers in their geographic proximity. These stakeholders were approached for the first time or had already expressed interest in participating in the national network of dementia care research. Stakeholders were contacted by email, phone, face‐to‐face or during other project presentations. Each potential partner received a cover letter communicating the project's objectives: establishment and cooperation within the TaNDem network and the purposes of the focus group interviews. For the focus group interviews, informed consent was obtained before the start. The Ethics Committee approved the TaNDem project of Greifswald University Medical School (Internal Reg. No.: BB 129/21).

### 2.2 Data collection

#### 2.2.1 The guided focus group Interviews

Since there was hardly any knowledge about the conceptual design, needs and expectations of health services research, health services practice and the required network structures and processes, such as preconditions and framework conditions as well as conducive and inhibiting factors for a national dementia care research network, and this was considered important for the establishment of sustainable structures, these key points were used as a basis for the design of the interview guide. The potential categories for the interview guide were previously extensively determined, discussed and selected by the responsible team of scientists and interviewers. The final categories represent the consensus reached by the expert group. The semistructured interview was introduced with a warm‐up question in which the interviewees could talk about their previous experiences with cooperation in existing networks. Furthermore, the semistructured interview guide consisted of the following categories: expectations, content, network infrastructure and implementation. This order was chosen to ensure an easy start by allowing all interviewees first to express their expectations, building on the expectations to define the more concrete contents of the network, to make statements about the implementation of these contents within a corresponding infrastructure and finally to determine the possible facilitating and hindering factors of implementation.

In addition to the main questions in each category, more detailed sub‐questions were formulated in advance, which could be asked during a break to stimulate the conversation further. An approximate time frame was set out for each main category so that as many questions as possible could be asked over 90 min. We asked all respondents to fill out a follow‐up contact form in case they wanted to participate in the online survey afterwards. The semistructured interview guide is shown in Appendix (Supporting Information: Table 1).

Each focus group interview included a minimum of 3 to a maximum of 15 people. The composition of the groups was not differentiated according to the respective field of work of the persons. Initially, focus group interviews were conducted using a structured interview guide. A total of 11 focus group interviews were conducted, 10 of which were conducted via the virtual platform ‘Lifesize’^23^ due to the contact‐restricting measures during the COVID‐19 pandemic and 1 interview in the presence at the Greifswald site with four participants. Following these focus group interviews, we conducted the quantitative part, prioritizing the responses from each topic area through an online survey.

Stakeholders were recruited with the help of around eight DZNE sites. Due to the division of labour, A.S., A.R., B.M., M.M.H. and D.P. from the DZNE Greifswald (eight interviews) and Witten (three interviews) conducted the focus group remotely online. The representatives of the other DZNE sites participated in these online focus group interviews but not as an interviewer. All interviewers have previous experience in conducting qualitative interviews.^24^, ^25^, ^26^, ^27^, ^28^ We ran the interviews from September to November 2021.

The interviews started with an introduction of the interviewers and a subsequent re‐explanation of the procedure and the network's goals to provide a common basis on which ideas can be developed. All participants were made aware of the voluntary nature of this interview and the possibility of interrupting or terminating the interview at any time. All participants were asked to comment on each question to reflect as much diversity of opinion as possible.

#### 2.2.2 The online survey

The guided focus group interviews were followed by the quantitative part, in which the responses from the respective topic areas were prioritized through an online survey. To quantify the priority of the preferences elicited from the guided interviews, an online survey was subsequently created using the tool ‘Lime Survey’.^29^ The survey included a selection of the three topics mentioned (content, network infrastructure and implementation), whose qualitative relevance was chosen through internal discussions within the research team. The first topic area, ‘expectations’, was not explicitly mentioned individually, as it was primarily used as a conversation starter and an opportunity for brainstorming. The statements in this area could very well be assigned to the ‘content’ category.

The codes that occurred most frequently per category were used for the online survey. Statements that occurred least frequently were not included in the online survey but were considered elsewhere in the design of the network. The survey aimed to rank the statements in each of the three categories to identify which thematic areas and collaboration factors are most important to the majority or should be implemented quickly.

We assigned topic‐specific subcategories to the three categories (i) content, (ii) network infrastructure and (iii) implementation to make the online survey clearer.

1. *Content*: Topics related to research and care, topics related to research, topics related to care, research and care offers, research offers and care offers.
2. *Network infrastructure*: Coordination and cooperation: types of meetings, coordination and cooperation: forms of digital cooperation.
3. *Implementation*: Conducive to successful cooperation, obstacles to successful cooperation.

The online survey took 5–15 min to complete. Using a drag‐and‐drop function, the interviewees could sort the statements according to their importance.

### 2.3 Data analysis

The interviews were audiotaped and subsequently transcribed by a transcription agency according to the semantic‐content transcription system based on Dresing and Pehl^30^ and then pseudonymized. The data analysis of the focus group interviews was based on Mayring's Qualitative Content Analysis^31^ using MAXQDA 2020.^32^ A coding manual was prepared using the deductive‐inductive mixed form of category formation.^33^ Based on the guideline, the three main categories are (i) content, (ii) network infrastructure and (iii) implementation and the categories (respective subquestions) could be named deductively in advance. Using the inductive procedure, subcategories could be added based on the answers to allow for a more detailed differentiation. A.S. and M.M.H. each coded their interviews at their own sites (Greifswald and Witten). The coding was done independently of each other. It was shared on the one hand because of the time component and on the other hand because the interviewers were most familiar with the interviews they had conducted themselves. After coding, all codes were merged using MAXQDA. Codes with similar meanings but different names were formulated uniformly at both sites with the help of the codebook (Supporting Information: Table 2). After about four rounds of discussion between A.S. and M.M.H., all codes were merged, and a common file was created.

We then conducted a descriptive analysis (frequency) of the online survey results using Microsoft®Excel.

## 3 RESULTS

The eight DZNE sites recruited approximately 300 healthcare providers and dementia researchers across Germany by email, telephone, in person or in the context of other project presentations. More than 150 stakeholders expressed interest in actively participating in the network via a letter of intent. All interested parties were informed about the network, and the upcoming focus group interviews, 87 of whom agreed to participate. The study's eligibility criteria were that the persons should either be healthcare providers focused on dementia or dementia (care) researcher. We recruited a total of 87 stakeholders from universities (9%), physician networks (5%), clinics (8%), dementia consultancies (46%) and the fields of nursing (9%), gerontology (6%), neurology (5%), occupational therapy (2%) and dementia research (10%). In addition to some participants with experience working in networks, others can make an important contribution through their expertise in their daily professional life. The field of activity of dementia counselling includes, for example, pointing out individual support options, strengthening the competence of relatives or raising awareness of the disease amongst doctors' practices, hospitals and home care services.

The focus group interviews showed stakeholders' perspectives, preferences and wishes regarding (i) content, (ii) network infrastructure and (iii) implementation of a participatory network for translational dementia care research. In addition, we collected information on the stakeholders' contribution to the network. The online survey quantified the results of the guided interviews and allowed for a ranking of the prior findings. A total of 76 participants out of 87 (87%) consented to follow‐up contact. We received the anonymized questionnaire from 54 participants (response rate: 71% of the people who had consented).

### 3.1 Relevant topics of the network about research and healthcare

Stakeholders saw an added value in the interaction of researchers and healthcare providers. They defined the transfer of results into practice as the most important goal of such a network (see Supporting Information: Table 3, row 1). In addition, the stakeholders would like to see a targeted and comprehensible presentation of support services (e.g., on homepages, flyers, information booklets) for those affected (e.g., self‐help groups or help with household management). Public relations work should provide information about diseases and raise awareness. Special attention should be paid here to marginalized groups that receive less attention (e.g., people with a migration background, and people with Down's syndrome) (Supporting Information: Table 3, row 2).

Stakeholders hope that this type of networking will lead to an intensification of care research, better access to data and PlwD for new studies, an investigation of regional differences in the care situation, greater involvement of relatives and an efficient assessment of dementia‐specific facilities (Supporting Information: Table 3, row 3). The majority wanted, above all, simplified access to research knowledge. In addition, the stakeholders demanded from the network a joint representation vis‐à‐vis politics and health insurance companies, support in the search for cooperation partners, the permanent availability of research funds and the emergence of a research consultancy in the sense of exchange between researchers (Supporting Information: Table 3, row 4). Furthermore, the respondents wished to develop strategies better to involve those affected, especially relatives, in the research. For example, new possibilities should be opened regarding how relatives can be co‐used as care professionals.

The prioritization results showed that care practice should focus as much as possible on providing support offers (e.g., in the form of information events and advice centres) for PlwD and caring relatives in a more accessible way. Other important topics were: the focus on individual, person‐centred care, the increase of contact points for early detection of dementia and the establishment of guidelines for the care of PlwD (Supporting Information: Table 3, row 5).

### 3.2 Preferred elements of the network infrastructure

The prioritization results showed that every third respondent (36%) favoured future regional networking through local face‐to‐face meetings. Most stakeholders (33%) could also imagine a network meeting of all members once or twice a year. However, just as many stakeholders (31%) were also prepared to participate in regular virtual working group meetings and thus facilitate a simple, supraregional exchange (Supporting Information: Table 3, row 6).

Stakeholders also stated that a regular exchange could be ensured via a communication platform with a chat forum (30%). The latest information from the network could be made available to network members via a newsletter (29%). The communication option considered most important by the majority (40%) is a database that contains the latest research results. It can be accessed at any time (e.g., via the website) (Supporting Information: Table 3, row 7).

### 3.3 Factors mentioned as supportive or hindering network implementation

The respondents felt that respect, trust, transparency and the recognition of competencies are important for long‐term cooperation. Furthermore, common goals and principles should be defined in advance for a successful network implementation (ranks 2 and 3). Stakeholders rated the availability of regional network contacts as the most important factor in this context (rank 1). Successful cooperation is made easier when the tasks of the stakeholders in the network match their competencies and the participants recognize an added value for their work (Supporting Information: Table 3, row 8). It was also considered very important that principles and goals are drawn up jointly and at eye level and that a concrete plan for further action is set up in advance (Supporting Information: Table 3, row 9).

The main obstacles mentioned were personnel, financial, and time limitations of the stakeholders (Supporting Information: Table 3, row 10). Furthermore, it was emphasized that the network should avoid being anonymous and poor accessible for individuals. Moreover, the network should not duplicate existing structures (e.g., Local alliances for people with dementia, the Alzheimer Society). TaNDem should rather build on and complement existing network structures (Supporting Information: Table 3, row 11).

Regarding their contribution to the network, stakeholders can imagine acting as contact persons, helping to mediate between research and practice and providing support in terms of public relations. On the one hand, they see opportunities to use their input in the network for their workplace, and on the other hand, they also emphasize time limitations and resource capacities.

Examples of focus group interview results are listed in Table 1, and exemplary prioritization results are in Table 2.

**Table 1:** Results of the qualitative interviews: Examples of needs of stakeholders, preferred infrastructure and facilitating and inhibiting factors of cooperation in the network.

**Topics related to research** Networking, mediation and exchange between research and practice Support in the search for cooperation partners Transfer of research results into practice or simplified access to research knowledge Announcement of new funding calls or research funds Research advice Involving people with dementia and their relatives in research Identification of research needs in practice
**Topics related to healthcare** Regional comparison of care situations Evaluation of the efficiency of dementia‐specific facilities and measures Improvement of hospital discharge management Development of care standards/guidelines for the care of people with dementia Attention to less‐considered groups of people (e.g., dementia and migration) Support services for relatives Focus on person‐centred care Contact points for early diagnosis and local counselling centres Further training for professionals and information courses for people with dementia and relatives Establishment of evidence‐based care models in practice
**Preferred elements of the network infrastructure** Fixed regional and supra‐regional contact persons and responsible persons Annual (virtual) national and regional network meetings Communication platform or exchange forum for network members as well as information channels via newsletters Database with the latest studies and research results
**Factors mentioned as supportive of network implementation** Local, regional contact person Building trust and cooperation at eye level Division of labour between external and internal actors as well as agreement on the scope of tasks and competences Uniform representation vis‐à‐vis health policy Public communication/public relations, as well as information and social sensitization in the field of dementia (public relations) Joint definition of principles and common, concrete goals Added value recognizable for own work
**Factors mentioned as hindering the implementation of the network** Personnel, time and financial limits for participation Continuity of the network after the funding period Anonymity and inaccessibility of stakeholders Regional differences Duplication of structures Difficulties in recruiting stakeholders, people with dementia and relatives

**Table 2:** Results of the quantitative survey: Stakeholders' needs, preferred infrastructures and facilitating and hindering factors for cooperation in the network.

Topics research and care	%	Rank
Transfer of results into practice	43	1
Attention to less‐considered groups of people	32	2
Regional comparison of care situations	25	3
**Topics research**		
Intensification of care/provision research	37	1
Facilitated communication of data and persons for care research	32	2
Evaluation of the efficiency of dementia‐specific facilities	31	3
**Topics care**		
Support services for people with dementia and family carers	34	1
Contact points for early diagnosis	24	2
Focus on person‐centredness (individualized care)	21	3
Guidelines for the care of people with dementia	21	4
**Research and care offers**		
Collected presentation of offers	40	1
Informing/social sensitization in the area of dementia	35	2
Public relations	25	3
**Research offers**		
Simplified access to research knowledge	32	1
Representation vis‐à‐vis politics and health insurance funds	24	2
Support in the search for cooperation partners	23	3
Research advice (exchange between researchers)	22	4
**Care offers**		
Information courses and counselling centres for people with dementia and relatives	38	1
Dementia‐specific training for caregivers and care facilities	31	2
Offers to relieve the burden on relatives	31	3
**Coordination and cooperation: Types of meetings**		
Regional meetings (in presence)/local networking	36	1
Network meetings of all network members in presence with hybrid option (1–2 × per year)	33	2
Supraregional working group meetings (regular, virtual)	31	3
**Coordination and cooperation: Forms of digital cooperation**		
Database with the latest research results for network members (e.g., accessible via website)	40	1
Possibility for direct digital exchange (e.g., a chat forum for network members)	30	2
Newsletter for network members	29	3
**Conducive to successful cooperation** (only the three most important ranks mentioned)		
Permanent local contact person	18	1
Establishment of principles and common goals	16	2
Building trust and cooperation at eye level	14	3
**Obstacles to successful cooperation** (only the three most important ranks mentioned)		
Staffing or turnover	16	1
Lack of financial resources	15	2
Duplicate structures	14	3

## 4 DISCUSSION

This study explored stakeholders' views in the TaNDem network on important dementia care research topics, the nature of collaboration in a future dementia care research network, and supporting and hindering factors in implementing stakeholder and PlwD involvement in Germany. With the help of focus group interviews and subsequent prioritization of the results, we gained important insights about sustainable and effective cooperation and stakeholders' participation in all research levels within a dynamic, self‐determined network.

### 4.1 The nature of collaboration in a future dementia care research network

Regarding the type of cooperation, a regional reference is necessary for successfully implementing the TaNDem network. This can be through regional meetings or by assigning a local contact person. However, it also became clear that the stakeholders are willing to network virtually and supraregional, for example, through virtual working group meetings, web‐based communication platforms and databases.

The usefulness of PlwD, relatives and stakeholders' online networking in care practice facilitates communication, increases efficiency and benefits all participants.^34^ In TaNDem, an information and communication platform has been set up as a portal with a user area for all interested parties. Furthermore, a study database is to be launched to promote the transparency of health services research and its results, as well as a research transfer platform providing ready access to research findings and improving their use in practice. A participatory research platform will help promote the involvement of PlwD and their relatives in all phases of research projects and will allow for permanent online networking. In addition, a method centre is to be created that provides expertise in the methods of dementia‐related care research and thus supports the design and implementation of research projects in the network.

### 4.2 Future dementia care research topics

Results showed that stakeholders are interested in collaboration and brought different issues to the fore. Most importantly, this study provided an overview of what stakeholders hope to gain from a partnership with healthcare providers and dementia researchers. The researchers would like to see a cooperative exchange of research in the future, from which they hope for support in the search for cooperation partners, joint representation vis‐à‐vis politics and health insurance funds, as well as the permanent availability of research funds.

Topics prioritized include an increased involvement of PlwD and their relatives (e.g., people with a migration background, people living with Down syndrome) in research, person‐centred care and the relief of caring relatives.

Looking at the results of other papers that addressed the research priorities of dementia research, it becomes clear that the topics of early detection of dementia, support and involvement of relatives,^3^, ^9^, ^10^, ^11^ person‐centred care,^3^, ^10^ destigmatisation of the disease,^3^, ^10^ knowledge transfer into practice, evaluation of the efficiency of dementia‐specific measures,^3^, ^9^, ^10^ the development of care standards/guidelines for the care of PlwD,^9^ which emerged in this study, are consistent with the findings of the previous studies. Newly elicited preferences were the desired regional comparison of care situations, evaluation of the efficiency of dementia‐specific facilities, improvement of hospital discharge management, attention to marginalized groups (e.g., people with migration backgrounds, people living with Down syndrome), the establishment of evidence‐based care models in practice, further training for specialist staff as well as information courses and local counselling centres for PlwD and relatives. Compared to the German study by Kowe et al.,^3^ this additional information could be gained because we involved more participants in this study, which were distributed throughout Germany. Bringing together stakeholders distributed throughout Germany could, for example, address the desire for a regional comparison of care situations. In addition, due to the involvement of many stakeholders with a wide range of professional backgrounds, less frequently represented topics, such as the involvement of PlwD with a migration background or Down syndrome, could be put on the agenda.

### 4.3 Influencing factors while implementing the TaNDem network

Linking the TaNDem network to existing structures would be an important component of using existing competencies and experiences and avoiding duplicate structures. Furthermore, activities need to be planned and implemented well‐structured so that the resources of the participants and the network members—especially for the PlwD and their families—are used sensibly. To the full extent, they can draw added value from their participation. The results of this mixed‐method study are largely consistent with the findings of Thandi et al.,^12^ who developed strategies for successful cross‐network collaboration. These authors also concluded that maintaining communication through regular team meetings, clearly defining goals and building relationships based on trust are key factors for successful implementation.^12^, ^35^ Similarly, the study by Kowe et al.^3^ concludes that stakeholders want an equal, respectful and transparent collaboration at eye level with researchers.

### 4.4 Limitations

However, this study also has several limitations. When looking at the prioritization results, it becomes clear that the topics have similar values in some cases, and thus, no ranking can be formed. One limitation of the study is, therefore, that the respondents were forced to rank the topics, even in cases they considered several issues to be equally important. However, the prioritization is only a supplement to the qualitative results of the focus group interviews. It mainly serves to identify whether certain aspects have been given a high priority across stakeholders.

Since participation in the focus group interviews was based on voluntariness, it is conceivable that mainly people who have already dealt with the topic more closely and tend to have a positive attitude towards cooperation have preferably participated, and thus the diversity of opinions of the care practice and research may not be fully represented.

A limitation of the mixed‐method study design is the possible loss of depth of the qualitative data through quantification.^36^ It cannot be ruled out that individual themes have been lost through the aggregation and rephrasing of the results of the focus group interviews. However, quantifying qualitative data is a complementary step in the evaluation to broaden the perspective and gather complementary views for interpretative analysis.^37^ Furthermore, due to the pseudonymization of the data, no conclusions could be drawn about the profession, regional differences or, for example, a person's age. For this reason, this work has a more quantitative than qualitative character. However, the promise of pseudonymizing personal data probably led to a higher likelihood of participation.

Furthermore, the online format may have created some limitations. Social interaction may be restricted by such a format, for example, by not being able to keep eye contact during the interviews or by only one person having the opportunity to speak at a time. However, in our case, these limitations should have little effect, as care was taken to ensure that all people said and had roughly similar amounts of time to speak. An attempt was made to avoid a limitation due to the interviewer effect by having only one person interview each of the two DZNE sites.

## 5 CONCLUSION

In summary, this study provides insights into which other, so far rather rarely researched but very essential, topics could be brought to the forefront of dementia care research. Likewise, to our knowledge, it becomes apparent which measures need to be taken to enable successful participatory collaboration and networking in the national context in Germany. If these measures are implemented, the TaNDem in Germany represents an opportunity to establish a link between the different fields, with significant potential to provide added value for all involved. The results form the basis for the further design and establishment of the network.

Furthermore, additional research is needed on methods and strategies for the successful participation of PlwD and their relatives in research,^15^ as well as stakeholders, to make research more dementia‐friendly and needs‐based. Furthermore, in future research, one could pursue an even stronger qualitative approach and analyze personal data (after prior consent) to evaluate information on regional differences, age, gender or occupational group.

## AUTHOR CONTRIBUTIONS

Annelie Scharf, Anika Rädke, Stefanie Köhler, Martina Roes, Stefan Teipel, Wolfgang Hoffmann and Bernhard Michalowsky made substantial contributions to the design of this study. Annelie Scharf, Anika Rädke, Daniel Purwins, Marie Marleen Heppner, Martina Roes and Bernhard Michalowsky made substantial contributions to the acquisition, analysis and interpretation of data. Annelie Scharf drafted the work, all remaining authors revised the work critically for important intellectual content. All authors approved this version to be published and agreed to be accountable for all aspects of the work in ensuring that questions related to the accuracy or integrity of any part of the work are appropriately investigated and resolved.

## CONFLICT OF INTERESTS STATEMENT

The authors declare no conflict of interests.

## Supporting information

Supporting information.

## Data Availability

The pseudonymised primary data used in this report are available on request from the corresponding author.
