# Supplementary material for: Design and implementation of the participatory German network for translational dementia care research (TaNDem): A mixed‐method study on the perspectives of healthcare providers and dementia researchers in dementia care research
Source: Health Expect. 2023 Mar 12;26(3):1009–18. doi: 10.1111/hex.13748 (PMC10154806; doi:10.1111/hex.13748)
Supplement: Supplementary file 1 — Supporting information. [file HEX-26--s001.docx]

**Appendix**

**Supplementary Table 1:** The semi-structured interview guideline (translated verbatim from german for the paper).

| **0: Experience (warm-up phase)**  I) What previous experience do you have with regard to cooperation in existing networks (in dementia-related health services research)? |
| --- |
| **A: Expectations**  I) What are your expectations of a network for dementia care research?   - What goals should such a network pursue? - Which topics (needs for research/needs for care) regarding care and research could be addressed with the help of a regional network? - Which actors would you like to work with within the network? - What do you see as the benefits of belonging to a network? |
| **B: Contents**  I) In your opinion, which topics/questions could/should be dealt with in a network for dementia care research?   - Whose topics (needs for research/needs for care) are in focus and why? (e.g. internal: (e.g. internal: needs of the network actors or external: societal needs of, e.g. local politics, other research institutions, practice partners in care). - Research, policy, exchange & communication, dissemination of results, addressees, offers.   II) Which offers could a network for dementia care research provide (local/regional/national/international)?  III) We have discussed your ideas on contents/offers that a network for dementia care research could work on: What are your three highest/for you priorities in this regard? Which ideas on content/offerings would be the most important? |
| **C: Network/infrastructure**  I) In your opinion, what structures (framework conditions) and processes are necessary so that the topics/issues you mentioned can be dealt with within a regional [national] network?  Think of...   - (if actors have already reported experiences in networks in the warm-up phase:) Which structures and processes would you consider useful - based on your experiences – about the network for dementia care research? - Actors - communication and cooperation among each other (e.g. how often do meetings occur?) - Meeting challenges (at the regional/national level) - Coordination and steering of the (regional/national) network - Decision-making within the network   II) We have discussed your ideas regarding structures and processes that a network for dementia care research should have: What are your three highest/for you priorities in this regard? Which ideas related to structures and processes would be the most important? |
| **D: Implementation**  I) What do you think is necessary to successfully implement the [regional/national] dementia care research network?   - Think of: gradual process, possible changes, criteria for success, generation of results. - Enabling factors - Hindering factors - risks   II) We have talked about your ideas for implementing the network: What are your three highest/for you priorities in this regard? Which arguments for implementation would be the most important?  III) What contribution would you be willing/able to make to a dementia care research network?   - Where do you see your role/responsibilities in the network? - What resources will you be able to provide if you participate in the network? - What would motivate you to participate in the establishment of such a network? |
| **E: Closing**  I) Are there still open points that you would like to mention / topics that have not been mentioned so far?   - Are there any other aspects/topics that could be relevant for the establishment and expansion of a network for dementia care research? |

**Supplementary Table 2:** The codebook (translated verbatim from german for the paper).

**Codebook**

| **1 RED quotes** | 10 |
| --- | --- |
| **2 Previous experience in existing networks** | 0 |
| 2.1 No / little previous experience in existing networks | 3 |
| 2.2 Previous experience in regional networks | 47 |
| 2.3 Previous experience in national networks | 7 |
| 2.4 Enabling factors of existing networks | 11 |
| 2.5 Hindering factors of existing networks | 45 |
| 2.6 Cooperation between different actors | 19 |
| **3 Contents** | 0 |
| 3.1 V. & F.: Questioning, topics, needs | 0 |
| 3.1.1 Networking and mediation between research and practice | 3 |
| 3.1.1.1 Promoting mutual interest and trust | 2 |
| 3.1.1.2 Collaboration for "practice-based" research | 5 |
| 3.1.1.3 Exchange between science and practice | 6 |
| 3.1.1.4 Understanding of conditions of research projects | 3 |
| 3.1.1.5 Transfer of results into practice | 16 |
| 3.1.1.6 Research support services for vDBs | 4 |
| 3.1.1.7 Data/people (access) for health services research | 5 |
| 3.2 F: Questions, topics, needs | 0 |
| 3.2.1 Intensification of nursing/care research | 5 |
| 3.2.2 Research needs related to dementia in minorities | 2 |
| 3.2.3 Regional comparison of care situations | 2 |
| 3.2.4 Considering the opinions of carers in surveys | 1 |
| 3.2.5 Effectiveness analysis of dementia-specific facilities | 1 |
| 3.2.6 Research on medication | 1 |
| 3.2.7 Research on new treatment options | 2 |
| 3.2.8 Research on the importance of participation, sport, social contacts | 1 |
| 3.2.9 Research on the importance of everyday structure | 1 |
| 3.2.10 Research on psychosocial care | 2 |
| 3.2.11 Measuring instruments for quality of life | 2 |
| 3.2.12 Needs-specific survey of PWDs | 1 |
| 3.2.13 Existence of research funds | 1 |
| 3.3 V: Questioning, topics, needs | 0 |
| 3.3.1 Attention to less noticed groups of people | 1 |
| 3.3.1.1 Support services for family carers | 6 |
| 3.3.1.2 Support services for people with early stage dementia | 7 |
| 3.3.1.3 Support services for PWD without relatives | 3 |
| 3.3.2 Guidelines/guidelines for the care of people with dementia | 3 |
| 3.3.3 Delirium prevention | 1 |
| 3.3.4 Assessment tools | 1 |
| 3.3.5 Assessment of needs of PWD and relatives | 4 |
| 3.3.6 Improving discharge management | 2 |
| 3.3.7 Focus on person-centredness (individual care) | 2 |
| 3.3.8 Bundling of services | 1 |
| 3.3.9 Addressing and destigmatising the disease | 1 |
| 3.3.10 Hardly any care support centres with a focus on dementia | 1 |
| 3.3.11 Lack of contact points for early diagnosis | 2 |
| 3.4 V. & F.: Services | 1 |
| 3.4.1 Informing/social awareness in the area of dementia | 0 |
| 3.4.1.1 National impetus to address specific issues | 2 |
| 3.4.1.2 local awareness raising in the area of dementia | 4 |
| 3.4.1.3 Regional dementia care map | 2 |
| 3.4.1.4 Access to (digital) information material | 2 |
| 3.4.2 Joint political action | 1 |
| 3.4.3 Public relations | 6 |
| 3.4.4 Collective presentation of services | 2 |
| 3.5 F: Offers | 0 |
| 3.5.1 Research advice: Exchange between researchers | 4 |
| 3.5.2 Simplified access to research knowledge | 18 |
| 3.5.3 Support in the search for cooperation partners | 1 |
| 3.5.4 Involving relatives in research | 1 |
| 3.5.5 Uniform representation vis-à-vis politics and insurance companies | 2 |
| 3.6 V: Services | 0 |
| 3.6.1 Expansion of care standards/ guidelines | 2 |
| 3.6.2 Free information brochures | 2 |
| 3.6.3 Establishment of case and care management | 1 |
| 3.6.4 Local contact points | 1 |
| 3.6.5 Relief for relatives | 3 |
| 3.6.6 Involvement of authorities | 1 |
| 3.6.7 Meetings for relatives | 1 |
| 3.6.8 Information courses for affected persons and relatives | 1 |
| 3.6.9 Personal assistance | 2 |
| 3.6.10 Counselling/training | 8 |
| 3.6.11 Staff development | 3 |
| 3.6.12 Expansion of financial resources | 1 |
| **4 Network/Infrastructure** | 0 |
| 4.1 Goals | 0 |
| 4.1.1 Improving health services research | 2 |
| 4.1.2 Participatory research | 0 |
| 4.1.3 Improving the care of PWD as a goal | 3 |
| 4.1.4 Openness to update existing information | 1 |
| 4.1.5 Pooling resources | 1 |
| 4.1.6 Uniform nationwide action | 3 |
| 4.1.7 Transfer of results into practice | 5 |
| 4.1.8 Better networking in rural areas | 2 |
| 4.1.9 Interprofessional networking | 4 |
| 4.1.10 Paying attention to less noticed affected groups of people | 2 |
| 4.1.11 Processing of scientific findings | 2 |
| 4.1.12 Support structures for Mmd and family members | 2 |
| 4.1.13 Adequate outpatient care | 3 |
| 4.2 Criteria for success | 0 |
| 4.2.1 Productive cooperation | 1 |
| 4.2.2 Publications | 1 |
| 4.2.3 Long-term establishment of the network | 1 |
| 4.2.4 Effective, sustainable results | 1 |
| 4.3 Benefit | 0 |
| 4.3.1 Practical benefit for Mmd | 1 |
| 4.3.2 Interprofessional exchange | 7 |
| 4.3.3 Information gain for practice | 10 |
| 4.4 Decisions | 0 |
| 4.5 Coordination & steering | 0 |
| 4.5.1 Network meetings | 10 |
| 4.5.1.1 Orientation of the network meetings to the work assignment | 3 |
| 4.5.1.2 Annual regular meetings (number) | 3 |
| 4.5.1.3 Virtual/present/hybrid meetings | 2 |
| 4.5.1.3.1 Excursus: Discussion on technology use among PWDs | 1 |
| 4.5.2 Permanent contact persons and responsible persons | 12 |
| 4.5.3 Low-threshold access to contact persons | 1 |
| 4.5.4 Organisation, content and structure of working groups | 6 |
| 4.5.5 Representation of interests / public relations | 1 |
| 4.5.6 Regional meetings | 3 |
| 4.5.7 Regular virtual exchanges | 10 |
| 4.5.8 Regional training on research results for stakeholders | 6 |
| 4.6 Communication & Collaboration | 0 |
| 4.6.1 Resources for & from stakeholders | 0 |
| 4.6.1.1 Time & energy of actors | 3 |
| 4.6.1.2 Competence & Expertise of Actors | 6 |
| 4.6.2 Knowledge/information exchange | 0 |
| 4.6.2.1 with PWD and relatives | 3 |
| 4.6.2.2 Practice and practitioners | 6 |
| 4.6.2.3 Research and research | 2 |
| 4.6.2.4 Research and practice | 6 |
| 4.6.2.5 regional and national | 4 |
| 4.6.3 Website | 6 |
| 4.6.4 Communication at eye level | 2 |
| 4.6.5 Database with latest research results | 5 |
| 4.6.6 Financial remuneration of network participants | 1 |
| 4.6.7 Newsletter | 6 |
| 4.6.8 Communication platform/ forum for stakeholders | 4 |
| 4.6.9 Local networking | 3 |
| **5 Implementation** | 0 |
| 5.1 Enabling factors | 0 |
| 5.1.1 Division of labour between external and internal actors | 3 |
| 5.1.2 Building trust | 5 |
| 5.1.3 Aspects of social encounter | 0 |
| 5.1.3.1 Cooperation without prejudice | 3 |
| 5.1.3.2 Cooperation at eye level | 8 |
| 5.1.3.3 Transparency of network/contact partners | 1 |
| 5.1.3.4 Recognition of all stakeholders | 3 |
| 5.1.4 With regard to cooperation | 0 |
| 5.1.4.1 Establishment of principles & common goals | 9 |
| 5.1.4.2 Regular concrete meetings to build the network. | 1 |
| 5.1.4.3 Involvement of stakeholders | 0 |
| 5.1.4.3.1 Informal/formal environment | 3 |
| 5.1.4.3.2 PWD and relatives | 8 |
| 5.1.4.3.3 Researchers | 1 |
| 5.1.4.3.4 Health care actors/sectors | 14 |
| 5.1.5 Public communication/public relations | 3 |
| 5.1.6 Openness to reformulation of objective(s) | 1 |
| 5.1.7 Concreteness in implementation | 5 |
| 5.1.8 Financial resources | 4 |
| 5.1.9 Matching the scope of tasks and competencies | 6 |
| 5.1.9.1 Fit with own work / interests | 1 |
| 5.1.10 Use of existing structures | 2 |
| 5.1.11 Involvement of authorities | 2 |
| 5.1.12 Local networking | 4 |
| 5.1.13 Clinical expertise | 1 |
| 5.1.14 Low-threshold approach | 2 |
| 5.1.15 Added value/benefit recognisable for individuals | 2 |
| 5.1.16 Local contact person | 3 |
| 5.1.17 Time-saving | 2 |
| 5.2 Hindering factors | 0 |
| 5.2.1 Inaccessibility | 1 |
| 5.2.2 Fluctuation | 3 |
| 5.2.3 Superficial individual interests | 2 |
| 5.2.4 Lack of feedback of research results into practice | 1 |
| 5.2.5 Recruitment of care actors, PWD & relatives | 6 |
| 5.2.6 Personnel limitations | 5 |
| 5.2.7 Insufficient orientation of NW meetings to the work mandate | 1 |
| 5.2.8 Continuation of the network after the funding period | 1 |
| 5.2.9 Anonymity | 1 |
| 5.2.10 Time intensity | 1 |
| 5.2.11 Lack of doctors | 2 |
| 5.2.12 Reaching those affected in a targeted manner | 1 |
| 5.2.13 Lack of political framework conditions | 1 |
| 5.2.14 Lack of financial resources | 3 |
| 5.2.15 Duplication of structures | 2 |
| 5.2.16 Mismanagement of finances | 1 |
| 5.2.17 Regional differences | 2 |
| 5.2.18 Uncertainty in funding by funding period | 2 |
| 5.2.19 Creation of necessary political conditions | 1 |
| **6 Own contribution** | 3 |
| 6.1 Active contribution | 3 |
| 6.1.1 Addition: Use synergy effects | 3 |
| 6.2 Serve as contact person | 2 |
| 6.3 Staff development | 1 |
| 6.4 Counselling/representation of relatives and PWDs | 1 |
| 6.5 Professional expertise as a resource | 1 |
| 6.6 Recruitment/intermediation of various actors Actors | 5 |
| 6.7 Own contribution to public effectiveness | 2 |
| 6.8 Promoting recognition of the care sectors | 2 |
| 6.9 Limitations of own contribution | 3 |
| **7 Attitude towards the network idea** | 1 |
| 7.1 Tendency towards a positive attitude | 10 |
| 7.1.1 Necessity of networking | 4 |
| 7.1.2 Extrinsic motivation | 2 |
| 7.1.3 Intrinsic motivation and/or fun | 7 |
| 7.1.4 Productive exchange as motivator | 2 |
| 7.1.5 Desire for recognition of practice in F. | 2 |
| 7.1.6 "it takes luck". | 1 |
| 7.2 Tending towards negative attitudes | 4 |
| 7.2.1 Criticism of the interview focus regarding the procedure | 2 |
| 7.3 Tendentially neutral attitude | 1 |
| 7.3.1 Uncertainties in practical implementation | 3 |

**Supplementary Table 3:** Meaningful quotes from the focus group interviews.

| **Category:** | **Subcategory:** |  | **Row** mentioned in the article |
| --- | --- | --- | --- |
| Relevant **topics** of the network about research and healthcare | Transfer of results into practice | "And I think the network could also do a lot of good by promoting understanding for health services research and knowledge about health services research in dementia patients. So not only knowledge about dementia and how to care for it better, but also why research is needed." [F1: 828-832]  "There are questions from those affected; they don't necessarily get answers. There are answers at the scientific level that don't necessarily reach the care system. That's why it would be important for me, especially through this network that you are thinking of, that there are results to questions from those affected, whose answers also reach those affected and whose answers also reach those who provide care and the professionals. Otherwise, we will have many, many results of good research that will remain somewhere in the air and will not reach where they are supposed to." [F7: 246-253] | 1 |
|  | Attention to marginalized groups | "We have cared for people with Down's syndrome and then said: We never had that on our radar. In the meantime, they are getting so old that they also develop dementia, which presents us with huge challenges. And, and there is simply nothing about that. So. That's how it was back then. Maybe there is something today, I don't know, but just things like that, where people come and say: Hey, that's what we are interested in right now." [F2: 616-622] | 2 |
|  | Regional comparison of care situations | "So you could now investigate what it's like in other regions in Germany. If I understand correctly, the network is set up nationally, so it would be interesting to see the regional differences because we know from other studies that there are very large regional, almost local differences, depending on the care situation. How is it in rural regions? No, we have only studied a mainly urban region here so that many follow-up questions could be formulated, which could only be dealt with in a network. We can't do that as a regional research institute." [F1: 441-450] | 3 |
|  | Research advice | "[...] I just call it a kind of singles' exchange now - that everyone writes a little bit: What can I do? Who do I have access to? So am I good at research? What kind of research do I do, or do I have access to patients or relatives? That you say a little bit: What do I offer? What am I looking for? What topics am I interested in? Where do I perhaps already have expertise? You don't just talk about people you already know, but also see: Okay, who else is there? Who could be suitable for the project or for the research question I have right now? I would find that quite interesting." [F5: 850-857] | 4 |
|  | Further training for professionals | "What I think is important is this training, that you expand this knowledge, that you can pass on the knowledge more and also prepare it in such a way. That I, let's say, get the information from this network, that I can then prepare it in such a way that I can give it directly to my inpatient nursing staff in the district, and the inpatient nursing staff then give it to the nursing staff so that it goes on and on." [F8: 594-599] | 5 |
| Preferred elements of the network **infrastructure** | Meetings in presence and online | "[…] now and then it can be online, and then it might even be good and important that you don't have to travel so far and save yourself a bit of time. But there also have to be face-to-face meetings where you can meet in person." [F2: 797-801] | 6 |
|  | Database | "I would like to see a dementia care research network also focus on ensuring that scientific findings reach the target group appropriately processed." [F4: 242-244] | 7 |
| Factors mentioned as supportive to network implementation | Regional contact person | "It would help enormously for this work if you know that people are coming to us at two different locations and you are already familiar with the contacts there." [F4: 839-841] | 8 |
|  | Cooperation at eye level | "You would have to create a forum where people really meet at eye level, just like now, no, so where you really - the practice meets the science and you just think together, so what is really important now?” [F2: 515-517]  "[…] what I find very, very important is that all areas are really represented, science and practice and people with dementia and relatives on the same level. That there is not one ranking, who is better, who is worse and who is more important, who has more to say, but everyone is important from his point of view, yes - for himself and the people he represents." [F2: 624-629] | 9 |
| Factors mentioned as hindering network implementation | Financial limits | "It is also a big issue is what happens next when the funding is no longer there when things are no longer financially supported." [F4: 883-885] | 10 |
|  | Duplicate structures | "...Because in the end, the motivation of the individual actors is that everyone has a benefit, which is most likely to come from the fact that we pool resources and use synergy effects and not everyone reinvents the wheel." [F5: 804-807] | 11 |
